# Predictors of Prolonged Grief Disorder in a German Representative Population Sample: Unexpectedness of Bereavement Contributes to Grief Severity and Prolonged Grief Disorder

**DOI:** 10.3389/fpsyt.2022.853698

**Published:** 2022-04-26

**Authors:** Bettina K. Doering, Antonia Barke, Anna Vogel, Hannah Comtesse, Rita Rosner

**Affiliations:** ^1^Clinical and Biological Psychology, Catholic University Eichstaett-Ingolstadt, Eichstaett, Germany; ^2^Clinical Psychology and Psychotherapy, Brandenburg Medical School Theodor Fontane, Neuruppin, Germany

**Keywords:** grief, bereavement, prolonged grief disorder, representative, risk factors, unexpectedness

## Abstract

Most people adapt to bereavement over time. For a minority, the grief persists and may lead to a prolonged grief disorder (PGD). Identifying grievers at risk of PGD may enable specific prevention measures. The present study examined the extent to which the subjective unexpectedness of the death predicted grief outcomes above and beyond known sociodemographic and objective loss-related variables in a sample drawn from a population-representative investigation. In our sample (*n* = 2,531), 811 participants (M_*age*_ 55.1 ± 17.8 years, 59.2% women) had experienced the loss of a significant person six or more months ago. Participants provided demographic and loss-related information, perceptions of the unexpectedness of the death and completed the Prolonged Grief Disorder-13 + 9 (PG-13 + 9). The PG-13 + 9 was used to determine PGD caseness. A binary logistic regression investigated predictors of PGD caseness, and a linear regression predictors of grief severity. ANCOVAs compared PGD symptoms between the groups who had experienced an “expected” vs. “unexpected” loss, while controlling for the relationship to the deceased and time since loss. The loss of a child (OR = 23.66; 95%CI, 6.03–68.28), or a partner (OR = 5.32; 95%CI, 1.79–15.83), the time since loss (OR = 0.99; 95%CI, 0.99–1.00) and the unexpectedness of the death (OR = 3.58; 95%CI, 1.70–7.69) were significant predictors of PGD caseness (Nagelkerke’s R^2^ = 0.25) and grief severity. Participants who had experienced the loss as unexpected (vs. expected) reported higher scores on all PGD symptoms. Unexpectedness of the death emerged as significant risk factor for PGD, even after controlling for demographic and other loss-related variables. While our findings replicate previous research on the importance of the relationship to the deceased as a risk factor for PGD, they also highlight the importance of assessing the subjective unexpectedness of a death and may help to identify risk groups who can profit from preventive interventions.

## Introduction

While bereavement through the loss of a loved one is a highly stressful life event, most people adapt to it over time without professional help (1–3). For a minority of the bereaved, however, grief does not abate and becomes what is termed prolonged grief disorder (PGD). ICD-11 (4) and DSM 5-TR (5) recognize PGD as a distinct mental disorder, though with slightly differing diagnostic criteria. A diagnosis according to ICD-11 requires the presence of at least one of two symptoms of separation distress (persistent and pervasive longing for the deceased or persistent and pervasive preoccupation with the deceased) and a least one of 10 accessory symptoms (e.g., difficulty accepting the death or feelings of guilt in relation to the loss). Additionally, these symptoms must persist to an impairing degree for at least six months after the loss and exceed social and cultural norms of grief. PGD according to DSM-5-TR requires the presence of at least one symptom of separation distress and at least three of eight accessory symptoms to the point of functional impairment in the last month. Additionally, the death of the close person must have occurred at least 12 months ago and symptoms must exceed social and cultural norms of grief (6). Prevalence estimates of PGD vary in accordance with the diagnostic criteria used and the sample under consideration: in recent meta-analyses, its estimated prevalence among bereaved persons ranged from 9.8% (7) to as high as 49%, when considering only persons bereaved by violent losses (8). In our representative study of the German population, the prevalence rate for PGD according to ICD-11 was 1.5% in the overall sample and the conditional prevalence (i.e., among persons having lost a significant other) was 4.2% (9). Compared to the cited meta-analyses, our estimated prevalence rate was relatively low. This could be due to the use of different classification systems (ICD-11 vs. various precursor concepts). More likely, however, differences in the samples under consideration play an important role: Lundorff et al. (7) stressed that most of the original studies included in their meta-analysis did not use a population-based approach with random-selection. Djelantik et al. (8) focused specifically on persons bereaved by unnatural losses. Both factors may be associated with higher rates of PGD than our population-based approach. The present manuscript focuses on PGD according to ICD-11 due to its international applicability and imminent implementation in the German health care system.

In order to inform PGD research and to offer preventive interventions more specifically to groups who are at an elevated risk for PGD and may profit from such treatments (10), it is crucial to identify the risk factors for PGD. Various empirical studies have investigated potential variables that contribute to the development of PGD [for reviews see (11–13)]. In their systematic review of the literature, Burke and Neimeyer (12) included studies in which variables were assessed longitudinally or clearly preceded the loss, were related to the death itself or could be assumed to have remained unchanged since the loss (e.g., gender). They grouped the risk factors into the categories “survivor’s background” (e.g., gender), “death- and bereavement-related” (e.g., cause of death), “relationship to the deceased” (e.g., kinship to the deceased), “intrapersonal” (e.g., neuroticism), “religion/belief” (e.g., worldview), and “interpersonal” (e.g., social support). Of all variables investigated, only six were identified as confirmed risk factors for higher grief intensity, i.e., examined in at least three studies and found statistically significant more than 50% of the time. The 32 other variables could only be established as potential risk factors as they did not meet these criteria. This clearly indicates the need for more research concerning the risk factors for PGD. In the present study, we focused on the perception of the death as unexpected as this has been relatively understudied, but could be targeted with an intervention. Ideally, a single study would include all potential predictors; however, this would overtax the participants by the sheer number of questionnaires. Therefore, for the purpose of the present investigation, we included three relatively well-established risk factors as control variables, i.e., female gender (12, 14–16), a closer relationship to the deceased [e.g., being a spouse or a parent (12, 14, 16, 17)], and shorter time since loss (8, 12, 14, 16). Additionally, given the range in our sample, we included age as a control variable. Concerning the age of the bereaved as a risk factor for PGD, meta-analyses and reviews report mixed findings, with two meta-analyses reporting non-significant findings (8, 16), one review reporting a significant negative association (12) and one meta-analysis a negative statistical trend [*p* = 0.075 (7)]. Our primary aim, however, was to examine the extent to which unexpectedness may have incremental validity as a predictor above and beyond the aforementioned variables.

Many studies have reported an elevated prevalence of PGD after sudden and violent losses (i.e., objectively assessed circumstances of the death) (8, 16, 18). In contrast, unexpectedness pertains to subjective experiences of the bereaved person. Perceived unexpectedness may contribute to PGD through several mechanisms. Perceived unexpectedness could hinder grief rituals such as saying good-bye that usually facilitate adaptation to bereavement (19). Unexpected deaths may lead to feelings of being less prepared for the death, which have been shown to be associated with PGD both concurrently and longitudinally (20). Unexpectedness could also increase difficulties in accepting the reality of the loss (21). Deaths that are perceived as unexpected are also less predictable. It has been proposed that since previous research demonstrates that humans prefer predictability, even when associated with negative events, unpredictability could negatively influence the grieving process (22). Based on these theoretical considerations, we expected that unexpectedness would be associated with a greater likelihood of PGD caseness and speculated that it would be more closely associated with certain PGD symptoms such as difficulties accepting the loss, disbelief or avoiding reminders of the loss.

Studies that have investigated the impact of subjective unexpectedness of the death (independently of the objective mode of death) on grief-related distress and PGD have yielded mixed results. A study of recent spousal bereavement reported no significant association between unexpectedness and bereavement outcome (23). In a similar vein, another study found that not being able to anticipate the death of a loved one did not influence pathological grief reactions (22). However, there is also evidence for an association between unexpectedness of the death and bereavement outcomes. Studies have reported that unexpectedness was associated with poorer bereavement outcome in spousal bereavement (24, 25). In samples of participants having lost a loved one due to an illness, greater perceived unexpectedness of the death was positively associated with poorer bereavement outcome (26) and higher PGD severity (27). Focusing on indicators of pathological grief, participants who reported an unexpected (vs. expected) loss reported higher PGD severity in a large Japanese epidemiological study (28). Unfortunately, the latter study excluded specific bereavements [i.e., exclusion of parents having lost a child (28)] and only investigated the respective bivariate relationship, which limits its generalizability. In a recent study focusing on the effects of bereavement during the COVID-19 pandemic, unexpectedness explained differences in pathological grief levels between other natural and COVID-19-related losses (29). Two large studies investigated unexpectedness as a risk factor for PGD severity (30) and a potential PGD diagnosis (31), respectively. In both studies, unexpectedness emerged as a significant predictor, even after controlling for the influence of sociodemographic and loss-related variables.

Unfortunately, the generalizability of many of these results remains limited. First, several studies used convenience samples (24, 27, 29, 30). Although instructive, using such samples cannot take into account the base rate of relevant factors in the general population. The associations between unexpectedness and PGD as reported in the individual studies may therefore be affected or biased by sampling effects. This is illustrated by a recent study which used consistent assessment methods and inclusion criteria across three different convenience samples of bereaved persons to investigate other risk factors for PGD: their general findings could not be reproduced across the samples (32). Second, few studies focus on associations between unexpectedness and PGD as a diagnostic category (28, 31). Most studies report results concerning associations with dimensional outcomes such as PGD severity (22, 27, 29, 30) or grief-related distress (23, 24, 26), respectively. Lastly, the studies used various instruments to assess PGD and PGD severity [e.g., Prolonged Grief-13 (33), Inventory of Complicated Grief (34), Brief Grief Questionnaire (35), Traumatic Grief Inventory Self-Report (36)], which are based on different underlying diagnostic concepts (e.g., prolonged grief disorder, persistent complex bereavement disorder or complicated grief). Since the final criteria for PGD according to ICD-11 have been established only recently, these studies share a common limitation from today’s viewpoint: they used various precursors of PGD as outcome and we do not know whether their results generalize to the present diagnostic concept of PGD. Thus, more research is needed concerning the association between unexpectedness of the death and PGD and dimensional grief severity according to ICD-11, respectively, in large, more population-representative samples (35, 37).

The first aim of the present study was to investigate the perceived unexpectedness of the loss as a risk factor for PGD caseness according to ICD-11 in a large sample of bereaved persons drawn from a population-representative study, while simultaneously controlling other risk factors such as sociodemographic variables (gender, age) and loss-related variables (relationship to the deceased, time since loss). We hypothesized that unexpectedness of the death would predict PGD caseness positively above and beyond the sociodemographic and loss-related variables (8, 12, 16). Since many of the previous findings regarding the association between unexpectedness and bereavement outcome relied on a dimensional approach [e.g., (24, 26, 27, 29, 30)], the second aim was to investigate these associations for grief severity. We expected that the same associations with the risk factors would hold true for grief severity as a dimensional variable. Lastly, we wanted to explore whether unexpectedness affects individual grief symptoms differentially.

## Materials and Methods

### Ethics

The institutional review board of the University of Leipzig (Germany) approved the study (145-19/ek, April 2nd, 2019). Potential participants received full information regarding the study purposes and procedures and provided written informed consent.

### Participants

This observational, cross-sectional study was conducted as part of a multi-topic survey concerning the physical and mental well-being of the German population, commissioned by the University of Leipzig. A sample, representative of the German population, was collected from May to July 2019. The sampling and data collection proceeded with help of a demographic consulting company (USUMA GmbH, Berlin, Germany).

Inclusion criteria for the multi-topic survey were age ≥14 years and sufficient German language skills. For the sampling, the area of Germany was divided into 258 sample areas representing the whole country. From these areas, households were selected by random route procedure. Within each selected household, one member who fulfilled the inclusion criteria was chosen via the Kish-selection technique. In total, 5,393 valid household addresses were contacted; 2,851 of those contacted failed to provide data for the following reasons: declined participation (household: 22.9%; target person: 12.3%), non-availability after four visits (household: 13.6%, target person: 3.0%), absence of target person (0.6%), and inability of the target person to follow the interview (0.5%).

With the remaining 2,542 participants, face-to-face interviews were scheduled. Trained interviewers (*n* = 219) informed the participants about the study aims and procedures, obtained written informed consent and collected sociodemographic data. Participants then completed the self-report questionnaire using paper-pencil versions. Interviewers were present until the participant indicated having completed the questionnaire and offered help if the participant did not understand the meaning of a question. Of the resulting 2,542 interviews, eleven could not be analyzed. The sample of the multi-topic survey consisted of 2,531 participants and was representative in comparison to the German micro census with regard to age, gender, and geographic region. The German micro census is a representative survey based on 1% of the German population [about 810,000 Germans (38)], which is used for political decision making in Germany.

Participants who reported the loss of a significant person six or more months ago and provided PG 13 + 9 data were eligible for the current study. Of the 2,531 participants from the multi-topic survey, 1,720 did not meet these inclusion criteria: not having experienced the death of a significant other (*n* = 1,584), not providing any answers for the PG13 + 9, although they indicated having suffered a loss (*n* = 33), not specifying the time since loss (*n* = 16) and time since loss below six months (*n* = 87). Thus, a total of 811 participants were included in the final sample for the present study. Table 1 provides the sample characteristics.

**TABLE 1:** Demographic and loss-related characteristics.

Variable	Frequency (%)	Mean (SD)	Valid n
**Demographic characteristics**			
Age	–	55.1 (17.8)	811
Gender		–	811
Men	331 (40.8%)		
Women	480 (59.2%)		
Education Group		–	790
Primary	554 (68.3%)		
Secondary	126 (15.5%)		
Tertiary	93 (11.5%)		
Other	17 (2.1%)		
Income Group		–	786
<1,250 Euro	308 (38.0%)		
1,250– 2,500 Euro	373 (46.0%)		
>2,500 Euro	70 (8.6%)		
No response	35 (4.3%)		
**Loss-related characteristics**			
Relationship; deceased person was		–	807
Parent	344 (42.4%)		
Partner	149 (18.4%)		
Child	28 (3.5%)		
Other family member	215 (26.5%)		
Friend	71 (8.8%)		
**Time since loss (months)**		113.2 (123.5)	811
Unexpectedness of the death		–	809
Unexpected	355 (43.8%)		
Expected	372 (45.9%)		
None/both	82 (10.1%)		

**FIGURE 1:**
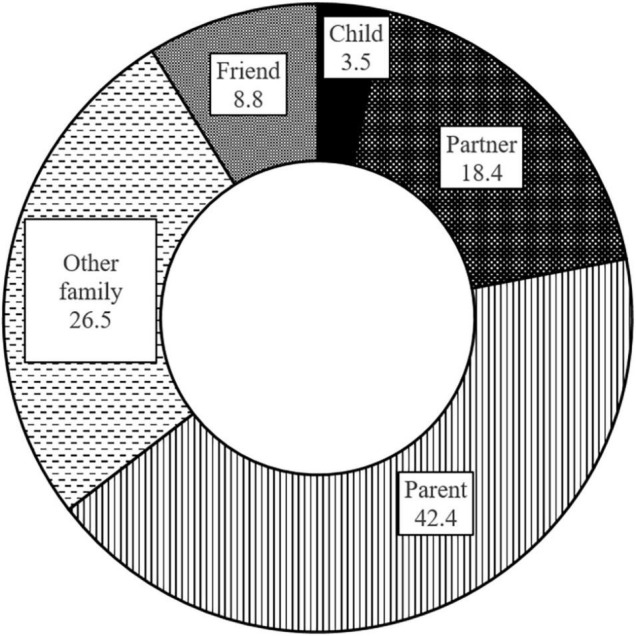
Relationship with the deceased person in percent.

Participant ages ranged from 14 to 95 years (mean: 55.1 ± 17.8) with 480 (59.2%) women and 331 men (40.8%). For education, income and further details see Table 1. The most common loss was death of a parent (42.4%) (Figure 1). The longest time since loss were 1055 months; with a mean of 113.2 ± 123.5 months. For 372 participants (45.9%) the loss was expected, for 355 (43.8%) unexpected and for 82 (10.1%) none/both; two participants did not respond to this item.

### Measures

Trained interviewers collected the sociodemographic information using a structured interview. Other data were obtained using self-report questionnaires.

#### Sociodemographic and Loss-Related Information

Participants provided information on age, gender, education, income and the loss they indicated having suffered including the relationship with the deceased and time since loss. To assess the perceived unexpectedness of the loss, they were asked: “How was your perception of the significant other’s death?” with the three answer categories “expected,” “unexpected,” or “none/both.”

#### Grief Symptoms

Grief symptoms were assessed with the German version of the Prolonged Grief Disorder-13 [PG-13; (33)], which was extended to cover all but one ICD-11 symptoms [the exception was “blame,” for a discussion see also (9, 39)]. The resulting PG13 + 9 [PG13 + 9; see (40)] was administered as a self-report questionnaire [for a detailed description see (9)]. Participants rated symptoms on a 5-point scale regarding their frequency (1 = not at all to 5 = several times a day) or intensity (1 = not at all, 5 = overwhelmingly). Grief-related impairment was rated dichotomously as present vs. absent (“Have the symptoms above caused significant impairment in social, occupational or other important areas of functioning?”).

##### Candidates for PGD Caseness

A full diagnosis of PGD can only be made based on a clinical assessment. However, we identified participants who are candidates for a diagnosis of PGD by examining whether participants fulfilled the diagnostic criteria on the basis of their PG13 + 9 answers. For the purpose of the categorical diagnosis, a symptom was treated as “present” if the participant scored ≥ 4 (i.e., present “at least once a day/quite a bit”) on the 5-point scale (33). Caseness was determined according to the ICD-11 diagnostic algorithm. The criteria are fulfilled if at least one symptom of separation distress and one or more accessory symptoms are present. The symptoms must be present for at least six months since the loss and be associated with functional impairment [for further detail see (9)]. In our previous study, we found that the diagnostic algorithms of PGD according to ICD-11 and according to DSM-5-TR demonstrated a very high pairwise agreement in our sample [κ = 0.87; 95% CI: 0.79–0.96 (9)] and only report the result for the PGD caseness according to ICD-11.

##### Grief Severity

Grief severity was calculated as the sum of the PG13 + 9 score for the items used to determine caseness, with the exception of the item assessing functional impairment. Referring to the German version of the PG13 + 9, this score therefore includes the items 1, 2, 6, 7, 10, 11, 14, 15, 17, 18, and 19 [see (9) for the item match to the ICD-11 criteria]. This resulted in a sum score (with a theoretical range of 11–121) which is used as an indicator for overall grief-related distress.

### Data Analysis

In order to test the basic relationships between grief severity and the sociodemographic and loss-related variables, we calculated Pearson correlations between the continuous variables grief severity, age and time since loss. We also calculated *t*-tests (respective Welch tests when the variances were not homogenous) for gender and unexpectedness of the death with the dependent variable grief severity. In order to test grief severity as a function of the relationship with the deceased, we computed an ANOVA followed by Bonferroni *post hoc* tests. As measures of effect size, correlation coefficients, Cohen’s *d* and η^2^ are reported.

Two regression analyses were performed with the predictors age, gender, relationship with the deceased, time since loss (in months) and unexpectedness of the loss. The predictors age and time since loss were entered as continuous variables. The nominal variables were dummy coded: Relationship with the deceased with the reference category loss of a parent and unexpectedness with the reference category expected loss. Both regression analyses were calculated blockwise with the method ENTER. In block 1 the demographic variables age and gender and in block 2 the loss-related variables relationship to the deceased and time since loss were entered. In block 3, unexpectedness of the loss was entered to examine whether it explained variance even after the other variables were included in the model. A logistic regression served to establish the predictors for PGD caseness: we calculated a binary logistic regression with the caseness as the criterion (PGD case/no PGD case). Odds ratios (OR) with 95% confidence intervals are reported. A linear regression examined the predictors for the criterion grief severity (as a dimensional score distinct from caseness).

To further investigate whether the participants who had experienced the loss as unexpected differed from those for whom the loss had occurred expectedly, we compared the groups with regard to their grief complaints (excluding participants who had answered “none/both” for expectedness). We used as dependent variables all symptom-level items of the PG13 + 9 included in ICD-11 and conducted a MANCOVA. In order to control for all other factors that contributed to the grief severity in the regression, they were entered as covariates. The MANCOVA was followed by Bonferroni corrected *post hoc* tests.

## Results

### Descriptive Analysis of the PG-13 + 9 and Bivariate Analyses

The mean PG13 + 9 score (including only the 11 items relevant for ICD-11) was 18.68 ± 8.24. According to the ICD-11 diagnostic algorithm, 38 participants (4.7%) were identified as PGD candidate cases. Among participants reporting the death as unexpected (*n* = 355), 7.0% were identified as potential PGD cases. In the group reporting the death as expected (*n* = 372), 2.7% were potential PGD cases.

In the overall sample, age correlated significantly with the time since loss (*r* = 0.19, *p* < 0.01) and grief severity (*r* = 0.09, *p* < 0.05). Grief severity and the time since loss were associated negatively (*r* = −0.16, *p* < 0.01). Women (19.3 ± 8.6) reported higher grief severity than men (17.7 ± 7.6; *t*(761.006) = 2.77, *p* = 0.006, *d* = 0.19). Grief severity was higher after an unexpected death (20.4 ± 8.9) than after an expected death [16.8 ± 7.3; *t*(685.420) = 5.94, *p* < 0.001, *d* = 0.44]. The ANOVA indicated significant differences for grief severity depending on the relationship with the deceased: *F*(4,802) = 24.94, *p* < 0.001, η^2^ = 0.111. Bonferroni *post hoc* tests showed that participants who had lost a child reported the highest grief severity (28.0 ± 9.8) compared to all other participants (all *p*s < 0.001). Participants who had lost a partner reported the second highest grief severity (22.5 ± 10.2) compared to all other participants except those who had lost a child (all *p*s < 0.01). Participants who had lost a friend (19.2 ± 7.1) showed a higher grief severity than those who had lost a parent (16.8 ± 7.1; *p* = 0.016).

### Prolonged Grief Disorder

The blockwise binary logistic regression with the categorical criterion potential PGD caseness showed that the predictors loss of child, loss of partner, and shorter time since loss contributed to the caseness (ΔR^2^ = 0.165). When these variables were included in the model, unexpectedness of the loss added a significant increment to the prediction (ΔR^2^ = 0.042). The final model explained 25% of the variance. For full detail, see Table 2.

**TABLE 2:** Blockwise hierarchical binary logistic regression with the criterion candidate for PGD caseness (PGD case/no PGD case).

	Wald (*df* = 1)	*p*	OR	OR 95% CI Lower	OR 95% CI Upper	ΔR^2^	χ ^2^	*df*	*p*
**Model 1^a^**						0.045	11.24	2	0.004
Constant	45.98	<0.001	0.007						
Age	9.54	0.002	1.035	1.013	1.057				
Gender^b^	0.41	0.523	0.798	0.398	1.598				
**Model 2**						0.165	43.05	5	<0.001
Constant	20.68	<0.001	0.019						
Age	0.44	0.506	1.009	0.983	1.036				
Gender^b^	0.06	0.810	1.096	0.518	2.318				
Loss of child^c^	27.35	<0.001	24.458	7.380	81.052				
Loss of partner^c^	11.28	<0.001	6.212	2.140	18.037				
Loss of other family member^c^	0.01	0.94	0.950	0.258	3.499				
Loss of friend^c^	0.20	0.657	0.615	0.072	5.240				
Time since loss	5.58	0.018	0.995	0.990	0.999				
**Model 3**						0.042	11.51	1	<0.001
Constant	26.22	<0.001	0.007						
Age	1.39	0.238	1.017	0.989	1.046				
Gender^b^	0.24	0.876	1.063	0.492	2.297				
Loss of child^c^	23.66	<0.001	20.297	6.034	68.280				
Loss of partner^c^	9.02	0.003	5.319	1.787	15.831				
Loss of other family member^c^	0.02	0.892	0.914	0.249	3.360				
Loss of friend^c^	0.76	0.382	0.382	0.044	3.308				
Time since loss	7.61	0.006	0.994	0.989	0.998				
Unexpectedness^d^	10.72	0.001	3.582	1.669	7.686				

*^a^χ^2^ values and p-values refer to the increments compared to the previous restricted models. The values for the whole models are as follows: Model 2: χ^2^ = 54.29, df = 7, p < 0.001, Nagelkerke’s R^2^ = 0.210; Model 3: χ^2^ = 65.79, df = 8, p < 0.001, Nagelkerke’s R^2^ = 0.252; Hosmer-Lemeshow-Tests for all models p > 0.20.*

*^b^Reference category: men.*

*^c^Reference category: loss of parent.*

*^d^Reference category: expected.*

### Grief Severity

The results for the linear regression predicting grief severity are presented in Table 3. The complete model [F(8,796) = 22.20, *p* < 0.001] explained 17% of variance, with each of the three blocks contributing a significant increment (Table 3) and the same predictors contributing to the grief severity as to the PGD caseness. In block three, unexpectedness explained an additional 4% of the variance in grief severity.

**TABLE 3:** Blockwise hierarchical linear regression with the criterion grief severity.

	*B*	SE	ß	*T*	*p*	ΔR^2^	F	*df*	*p*
**Model 1^a^**						0.017	6.94	2, 802	0.001
Constant	13.90	1.31		10.60	<0.001				
Age	0.04	0.02	0.087	2.47	0.014				
Gender^b^	1.60	0.59	0.096	2.73	0.006				
**Model 2**						0.128	23.76	5, 797	<0.001
Constant	16.90	1.41		12.01	<0.001				
Age	0.002	0.02	0.004	0.11	0.910				
Gender^b^	0.93	0.56	0.055	1.66	0.096				
Loss of child^c^	11.52	1.51	0.256	7.62	<0.001				
Loss of partner^c^	5.07	0.83	0.238	6.12	<0.001				
Loss of other family member^c^	0.23	0.70	0.013	0.33	0.739				
Loss of friend^c^	1.70	1.01	0.059	1.69	0.092				
Time since loss	–0.01	0.002	–0.181	–5.29	<0.001				
**Model 3**						0.038	36.98	1, 796	<0.001
Constant	15.12	1.41		10.74	<0.001				
Age	0.02	0.02	0.032	0.82	0.413				
Gender^b^	0.99	0.55	0.059	1.81	0.071				
Loss of child^c^	10.60	1.49	0.236	7.13	<0.001				
Loss of partner^c^	4.70	0.81	0.221	5.78	<0.001				
Loss of other family member^c^	0.10	0.69	0.006	0.15	0.880				
Loss of friend^c^	0.32	1.02	0.011	0.32	0.750				
Time since loss	–0.01	0.002	–0.211	–6.23	<0.001				
Unexpectedness^d^	3.36	0.55	0.203	6.07	<0.001				

*^a^F values and p-values refer to the increments compared to the previous restricted models.*

*^b^Reference category: men.*

*^c^Reference category: loss of parent.*

*^d^Reference category: expected; Adjusted R^2^ for each model: Model 1: R^2^_adjust_ = 0.015; Model 2: R^2^_adjust_ = 0.137; Model 3: R^2^_adjust_ = 0.174.*

**FIGURE 2:**
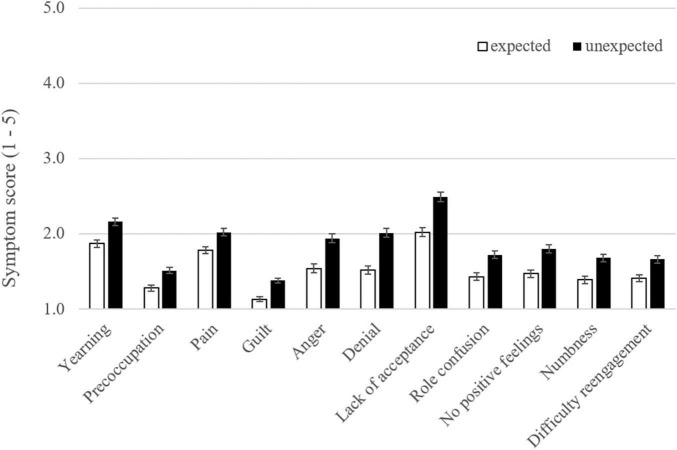
Mean scores of grief symptoms as a function of the expectedness of the loss.

In order to investigate the association between unexpectedness and the individual symptoms, we grouped the participants according to the unexpectedness of their loss (expected vs. unexpected). We then compared the mean severity of individual symptoms between groups with a MANCOVA, entering the variables that were associated with grief severity in the regression (relationship with the deceased, time since loss) as covariates. The MANCOVA yielded effects for the covariates [relationship with the deceased: *F*(11, 696) = 11.02, *p* < 0.001, η_*p*_^2^ = 0.148; time since loss: *F*(11, 696) = 7.31, *p* < 0.001, η_*p*_^2^ = 0.104] and a main effect for the group [*F*(11, 696) = 6.98, *p* < 0.001, η_*p*_^2^ = 0.099]. Bonferroni corrected *post hoc* tests revealed that even after controlling for the relationship with the deceased and the time since loss, the participants who had experienced the loss as unexpected reported higher symptom scores with respect to every individual symptom (Figure 2). The effect sizes of all comparisons were small to medium (Cohen’s *d*: 0.25–0.45).

## Discussion

The present study examined the extent to which the subjective unexpectedness of the death predicted grief outcomes above and beyond known sociodemographic and objective loss-related variables. In our sample drawn from a German population-representative study (9), the following variables significantly increased the likelihood of PGD caseness according to ICD-11: loss of a child or a partner, shorter time since loss and, even when controlling for the influence of all other predictors, perceived unexpectedness of the death. The same variables were also predictors of higher grief severity, when using a dimensional approach. On a single symptom level, the group of participants who had experienced the death as unexpected reported higher scores for each PGD symptom. Our results therefore highlight that in addition to objective risk factors, subjective experiences associated with the circumstances of the death, such as unexpectedness, are important risk factors for PGD.

Our main interest was examining the extent to which subjectively experiencing the death as unexpected was predictive of PGD and grief severity after taking into account known objective predictors. Our results demonstrate that unexpectedness is associated with an elevated risk of PGD and grief severity. This is in line with results from previous studies that have focused on the association between unexpectedness and grief severity (27, 29, 30) and potential PGD caseness (31), respectively. However, it extends this finding to a population-representative sample [in contrast to (27, 29)]. Importantly, and complementing large population-based samples, our study focuses on unexpectedness as a predictor in and of itself [in contrast to combining it with cause of death (41)] and demonstrates the incremental value of unexpectedness as a predictor over and above other sociodemographic and loss-related variables [in contrast to Deno et al. (28)].

It is interesting to note how widespread the perception of unexpectedness of death was in our sample. This mirrors the results from previous studies, which also reported a high number of deaths perceived as unexpected (28, 31): to illustrate, in a convenience sample of 241 bereaved participants, 55% of the sample reported having experienced the death as unexpected, even though 77% of the same sample reported the cause of death as natural (30). Unexpectedness of the death is a risk factor that will probably become even more relevant in the wake of the COVID-19 pandemic, since COVID-19 related deaths are likely to be perceived as unexpected (42). A recent study investigated acute COVID-19 related bereavement vs. bereavement by other natural or unnatural causes (29). COVID-19 related deaths were indicated more frequently as unexpected (63 vs. 28% after natural deaths) and unexpectedness mediated the effect of cause of death on the acute grief levels of the participants. Since 28% of the natural deaths were experienced as unexpected, these findings also highlight the importance of separating unexpectedness as a subjective perception from the objective cause of death rather than inferring one from the other.

We also investigated whether unexpectedness vs. expectedness of the death affected manifestations of PGD on the symptom level in a bereaved sample. We did not find a specific PGD symptom profile for unexpected losses as has been reported in a sample of bereaved parents (43). This discrepancy could be due to differences in the samples under consideration (representativeness, time since loss, age, and gender) or the different instruments used [Inventory of Complicated Grief (34) vs. PG13 + 9 ICD-11 algorithm]. Future harmonization of the assessment instruments and criteria could certainly help to interpret such discrepancies with more confidence (44). However, participants who indicated that they had suffered an unexpected loss reported higher scores on every single symptom of PGD with small to medium effect sizes. It is important to note that this analysis was not limited to participants meeting the diagnostic criteria of PGD. Its results therefore indicate that unexpectedness is associated with higher grief severity in the absence of a clinical diagnosis (even years after the loss).

One of the most well-established predictors for PGD in the literature is the relationship with the deceased (12, 16). Especially the loss of a child can increase the risk for PGD, as demonstrated across different cultures (14, 45–47). Compared to other losses, losing a spouse or a child seems to convey a considerable risk for PGD [e.g., (14, 47)]. This association was also evident in our analysis, with the loss of a child as the most influential risk factor. Another well-established risk factor is shorter time since loss (8, 12, 16), although a recent meta-analysis did report a non-significant association (7). In accordance with another study that was also based on a German population-representative sample (14), we found that shorter time since loss was a risk factor for PGD among participants whose loss dated back at least 6 months. However, the effect size of the association between time since loss and PGD was relatively small in our sample as well as in other studies [e.g., (14)] and must therefore not be overstated in its importance.

Gender was not a significant predictor for PGD. Our finding concerning gender is somewhat surprising, since being female is considered a well-established risk factor for PGD (12, 14–16). Recent meta-analyses, however, also reported no significant effect of gender on PGD prevalence both after natural and unnatural losses (7, 8). The contradictory findings could be due to the use of different measurement instruments and criteria for pathological grief in the original studies. Additionally, the typical overrepresentation of females in bereavement research (48) may complicate the investigation of gender effects in convenience samples and thus contribute to divergent results. Reflecting the general reliance on convenience samples in the original studies, the meta-analyses are also partly based on studies from convenience samples. Population-representative samples are therefore uniquely relevant to investigate this effect. Interestingly, our finding that gender did not predict PGD is in contrast to the other population-representative studies on PGD conducted so far (14, 41). Among the population representative studies, however, ours is the first to use a diagnostic algorithm of PGD according to ICD-11. It is therefore possible that ICD-11 criteria are less prone to gender effects than previous algorithms. Support for this line of argument comes from a recent registry-sampled cohort study in spousal bereavement (49): using the new ICD-11 criteria and growth-mixture modeling, Lundorff and colleagues demonstrated that all trajectories of prolonged grief comprised similar proportions of men and women. Another recent study, however, reported female gender as a risk factor for PGD according to ICD-11 in three convenience samples (32). Clearly, more research is needed to evaluate the association between gender and PGD according to the current ICD-11 conceptualization.

In our sample, the age of the bereaved person was not a risk factor for PGD, although our sample covered a broad range of ages (14–95 years). While this finding is in contrast to a previous review reporting younger age as a risk factor for more grief-related distress (12), it is in accordance with recent meta-analyses (7, 8). Comparing our result to the other study using a sample drawn from German population-representative sample, the latter study reported older age as a risk factor for PGD (14). Kersting et al., however, investigated the association between age groups and PGD prevalence, not age as a continuous variable and found that participants aged over 61 years were more likely to experience PGD as compared to other age groups. Importantly, the study used age groups with broad spans e.g., grouping participants in the age range from 61 to 94 years into one category. The present study used age as a continuous variable. This is an important difference between the two analytical approaches and possibly affects the results. In addition,—as discussed above with regard to gender—the diagnostic criteria for PGD also differ between the studies. Another possibility is that the relationship between age and PGD could be moderated by circumstances of the bereavement (e.g., kinship with the deceased and time since loss). For example, losing a parent in childhood may be associated with a different bereavement outcome at older age than having experienced the same loss in adulthood. Unfortunately, our study was not powered to assess such moderating effects.

### Strengths and Limitations

Strengths of our study are the large sample drawn from a population-representative survey, the use of a well-validated diagnostic instrument to assess PGD with a mapping of the items onto ICD-11 criteria [see (9)] and our analysis spanning cases, syndrome and symptom levels. At the same time, certain limitations must be acknowledged. First, while the sample of bereaved persons was adequately large, the number of potential PGD cases in the sample was lower than reported in previous studies [e.g., (14)]. Our use of ICD-11 criteria and a diagnostic algorithm instead of a cut-off score may explain this discrepancy. Additionally, we cannot make any assumptions with regard to the representativeness of our sample for the bereaved population even though many subgroups of the bereaved were present in our sample (e.g., bereaved parents, widowed persons). The robustness of the risk factors and the generalizability of our results therefore need to be confirmed by further studies. Second, we focused on the subjective experience of the circumstances of the death and did not assess more objective indicators, such as the cause of death. Naturally, the cause of death and the experience of unexpectedness are sometimes interrelated: losses resulting from sudden and violent modes of death (i.e., suicide, homicide, or fatal accidents) are more likely to be experienced as unexpected. Nevertheless, objectively “sudden” deaths, such as suicide, can be experienced as expected [e.g., 46% of participants bereaved by suicide rated the death as expected (50)]. Natural deaths can also be perceived as unexpected (26, 29, 51). Thus, these subjective and objective parameters should not be equated and are each worth consideration. Additionally, a recent investigation corroborates that unexpectedness and cause of death contribute independently to PGD symptoms (52). Therefore, it would be worthwhile to replicate our study with cause of death as an additional predictor. Future studies could also use a continuous measure of unexpectedness. Importantly, our correlative design and retrospective judgment of the unexpectedness cannot exclude that the grief severity affected the reports regarding the perceived unexpectedness. Reporting the death as unexpected may be a consequence of PGD symptoms, rather than a cause of PGD. Nonetheless, reporting that the death was unexpected could still be a useful indicator of the likely presence of PGD. Longitudinal studies could assist in clarifying this relationship. Lastly, while we used a well-established measure of PGD, our PGD assessment was based on a self-report and not a clinical interview by a trained psychologist. The diagnosis of PGD cannot be established on self-report data alone.

### Future Research and Implications

Our results have implications for clinical practice and future research. More knowledge about the risk factors for PGD can help to improve the identification of bereaved persons at risk for PGD and to refine grief-specific interventions. Stepped-care approaches or tiered models of bereavement care become increasingly recognized as helpful concepts to guide the development and allocation of support services (53, 54). In order to target and tailor these interventions, reliable information about who is at risk for PGD is necessary. Few previous studies investigating risk factors have used the present ICD-11 criteria for PGD. Our study shows that while some well-established risk factors (e.g., losing a child or a partner) apply also to PGD according to its present definition, other risk factors (such as gender) may need to be re-examined. Future research is needed that uses well-established diagnostic instruments for PGD in its present form and investigates large representative samples and oversamples participants with clinically relevant PGD symptoms. Our data also highlight the potential value of assessing the subjective unexpectedness of the death. Future research on risk factors in PGD that incorporate assessments of objective circumstances of the death and subjective experiences will further our understanding of PGD.

Taken together, our study identified unexpectedness of the death, relationship to the deceased and time since loss as risk factors for PGD according to its current diagnostic criteria (ICD-11) in a sample drawn from a population-representative study. The present findings call into question some previously established risk factors for PGD (e.g., gender), while at the same time corroborating evidence for others (e.g., relationship to the deceased). They also highlight the importance of the perceived unexpectedness of the death as a risk factor for PGD and elevated grief-related distress. This finding is especially important with regard to the current challenges that bereaved persons face during COVID-19 and the identification of persons in need of additional bereavement support.

## Data Availability Statement

The raw data supporting the conclusions of this article will be made available by the authors, without undue reservation.

## Ethics Statement

The studies involving human participants were reviewed and approved by Institutional Review Board, University of Leipzig, Germany. Written informed consent to participate in this study was provided by the participants’ legal guardian/next of kin.

## Author Contributions

RR obtained funding. BD and AB undertook the statistical analyses. BD and AB wrote the complete draft of the manuscript, AV, HC, and RR critically revised the earlier versions of the manuscript. All authors designed the study and contributed and approved the final manuscript.

## Conflict of Interest

The authors declare that the research was conducted in the absence of any commercial or financial relationships that could be construed as a potential conflict of interest.

## Publisher’s Note

All claims expressed in this article are solely those of the authors and do not necessarily represent those of their affiliated organizations, or those of the publisher, the editors and the reviewers. Any product that may be evaluated in this article, or claim that may be made by its manufacturer, is not guaranteed or endorsed by the publisher.
